# Development of a spontaneous pain indicator based on brain cellular calcium using deep learning

**DOI:** 10.1038/s12276-022-00828-7

**Published:** 2022-08-18

**Authors:** Heera Yoon, Myeong Seong Bak, Seung Ha Kim, Ji Hwan Lee, Geehoon Chung, Sang Jeong Kim, Sun Kwang Kim

**Affiliations:** 1 Department of Physiology, College of Korean Medicine, Kyung Hee University, Seoul, 02447 Republic of Korea; 2 Department of Science in Korean Medicine, Graduate School, Kyung Hee University, Seoul, 02447 Republic of Korea; 3 Department of Physiology, Seoul National University College of Medicine, Seoul, 03080 Republic of Korea; 4 Department of Biomedical Sciences, Seoul National University College of Medicine, Seoul, 03080 Republic of Korea

**Keywords:** Pain, Fluorescence imaging

## Abstract

Chronic pain remains an intractable condition in millions of patients worldwide. Spontaneous ongoing pain is a major clinical problem of chronic pain and is extremely challenging to diagnose and treat compared to stimulus-evoked pain. Although extensive efforts have been made in preclinical studies, there still exists a mismatch in pain type between the animal model and humans (i.e., evoked vs. spontaneous), which obstructs the translation of knowledge from preclinical animal models into objective diagnosis and effective new treatments. Here, we developed a deep learning algorithm, designated AI-bRNN (Average training, Individual test-bidirectional Recurrent Neural Network), to detect spontaneous pain information from brain cellular Ca^2+^ activity recorded by two-photon microscopy imaging in awake, head-fixed mice. AI-bRNN robustly determines the intensity and time points of spontaneous pain even in chronic pain models and evaluates the efficacy of analgesics in real time. Furthermore, AI-bRNN can be applied to various cell types (neurons and glia), brain areas (cerebral cortex and cerebellum) and forms of somatosensory input (itch and pain), proving its versatile performance. These results suggest that our approach offers a clinically relevant, quantitative, real-time preclinical evaluation platform for pain medicine, thereby accelerating the development of new methods for diagnosing and treating human patients with chronic pain.

## Pain: AI-based platform could aid analgesic drug discovery

A microscopy technique coupled with an artificial intelligence (AI) platform could help researchers discover new types of pain-relief medicines. A team from South Korea led by Sun Kwang Kim of Kyung Hee University and Sang Jeong Kim of Seoul National University created a machine-learning algorithm that converts calcium signaling data in the brain, as estimated via imaging on genetically engineered mice, into a measurement of pain intensity. The researchers applied the technique to several mouse models of chronic pain and showed that it accurately captured the analgesic effects of known painkillers. They also extended the system to multiple brain regions, cell types and another brain-controlled sensory process, itch. The researchers propose using the AI-based tool to evaluate candidate anti-pain and anti-itch medicines ahead of human trials.

## Introduction

Spontaneous ongoing pain is a primary complaint in patients with chronic intractable pain^1^. It leads to a heavy financial burden on society, affects the daily life of patients, and increases their suicide rates^2^. Conventional analgesics for chronic pain are frequently associated with severe side effects^3^. For example, opioids, a widely prescribed class of drugs for chronic pain, are most commonly used in the United States, where they have caused health crises such as addiction, overdose, and even death^4,5^. Recently, opioids have been an inevitable component of pain treatment in COVID-19 patients, raising concerns about side effects^6^.

Numerous non-opioid analgesics and diagnostic methods have been suggested based on a growing understanding of the mechanisms of pain in animal models. However, few have been successfully translated into clinical interventions^7–9^. A mismatch in pain type between humans and animals (i.e., spontaneous versus stimulus-evoked pain) has been claimed to be a major reason for these failures^10^. Many challenges exist in the objective assessment of spontaneous pain in animal models as well as human patients, and researchers have typically measured stimulus-evoked pain instead of spontaneous pain due to this restriction^11^. The lack of quantitative measurement techniques for spontaneous pain has impeded the translation of a vast amount of preclinical knowledge into clinical diagnosis and treatment.

Although several methods have been developed in preclinical contexts to overcome this issue, they have inevitable limitations. The grimace scale (GS) identified spontaneous pain in rodents based on their facial expressions^12^. However, the GS could not be applied to animal models of subchronic or chronic pain, including persistent inflammatory or neuropathic pain. Another method, the conditioned place preference (CPP) paradigm, can assess the presence of spontaneous neuropathic pain and analgesic effects by associating pain relief with a place that has a specific environmental cue^13^. However, this method is time consuming and requires the pharmacokinetics and administration route of the drug to be considered. In addition, drugs that have an impact on learning and memory or reward systems are limited with CPP^14^. Thus, there is an unmet need for a new methodology to objectively quantify spontaneous pain and the effects of painkillers.

The neuronal processing of pain information involves multiple brain areas. Among these, the primary somatosensory cortex (S1) plays a key role in the perception and discrimination of pain sensation by encoding its intensity, location, and temporal course^15–17^. This led us to hypothesize that neuronal activity patterns in the mouse S1 differ between spontaneous pain and non-pain conditions and that spontaneous pain can be measured quantitatively based on this discrepancy. Neurons in the S1 receive and discriminate various forms of somatosensory input, including touch, itch and pain^17–20^. This complexity makes it difficult to extract pain-specific information from neuronal activity patterns. Hence, to detect spontaneous pain information from noise-polluted signals in S1, we employed a bidirectional recurrent neural network (bRNN), a deep learning method specialized in time-series data analysis^21^. Furthermore, we optimized the preprocessing steps (Average training and Individual test, AI) for supervised learning from biology-driven data. The consequent deep learning model, designated AI-bRNN, successfully classified spontaneous pain in various pain models with different prognoses and evaluated the effects of various analgesics. Additionally, we demonstrated that AI-bRNN is versatile, performing well for other brain areas and cell types as well as other types of somatosensation.

## Materials and methods

### Experimental animals

All mice were C57BL/6 males aged 5–6 weeks old at the start of the experiments. The mice were housed in groups of two to minimize stress. The vivarium was controlled with a 12/12-h light/dark cycle, and all experiments were performed during daylight hours. All experimental procedures were approved by the Seoul National University Institutional Animal Care and Use Committee and performed in accordance with the guidelines of the National Institutes of Health.

### Behavior test

The formalin test was performed in mice that had been individually exposed to the observation chamber. Before the test, the mice were acclimated to the chamber for 1 h on 3 consecutive days. Ten microliters of 5% or 1% formalin solution was injected into the right hind paw; the mouse was then immediately put back into the chamber. We recorded the time spent in nociceptive behavior (licking and biting of the injected paw) every 5 min. In the experiments involving analgesic drugs, ketoprofen (100 mg/kg, 50 μl, i.p.) or 2% lidocaine (10 μl, subplantar, s.c.) was administered 20 min before the formalin injection. To quantify the relieving effect of gabapentin/venlafaxine (GB/VX) on partial sciatic nerve ligation (PSL)-induced neuropathic mechanical allodynia, the mice were placed in a 12 (d) × 8 (w) × 6 (h) cm clear plastic cage on a metal mesh. Behavioral tests were performed 30, 60, and 120 min after GB (100 mg/kg) and VX (50 mg/kg) administration. Mechanical allodynia was assessed using the von Frey filament (Linton Instrumentation, Norfolk, UK). Specifically, von Frey filaments delivering different bending forces (2.36, 2.44, 2.83, 3.22, 3.61, 3.84, 4.08, and 4.31 expressed as the log of the bending force in mg) were applied to the right hind paw using the up-down method, and the threshold force corresponding to 50% withdrawal was determined^22^. The experimenters were blinded to the treatments that the animals had received.

### Surgical preparation for imaging in awake mice

All surgical procedures were performed under isoflurane anesthesia (1–1.5%). To minimize edema and related inflammation, dexamethasone (0.2 mg/kg) and meloxicam (20 mg/kg) were administered by subcutaneous injection. A cranial window was made above the area of the left S1 corresponding to the hind paw (size: 2 × 2 mm; center relative to bregma: lateral, 1.5 mm, posterior, 0.5 mm)^23,24^. A small craniotomy was carefully performed using a #11 surgical blade. The exposed cortex was perfused with artificial cerebrospinal fluid, and an adeno-associated virus expressing GCaMP6s (AV-1-PV2824; produced by the University of Pennsylvania Gene Therapy Program Vector Core) was injected into S1 (30–50 nl per site; 200–300 μm from the surface) using a broken glass electrode (20–40 μm tip diameter). After virus injection, the exposed cortex was covered with a thin cover glass (Matsunami, Japan), and the margin between the skull and the cover glass was tightly sealed using Vetbond (3 M) and dental cement. For imaging of the ipsilateral cerebellar Bergmann glia, a small craniotomy was performed over lobule IV/V of the cerebellar vermis. AAV5.GfaABC1d.Lck-GCaMP6f was delivered to the cerebellum (100–300 μm beneath the surface) as described above.

### Two-photon Ca^2+^ imaging in awake mice

For awake imaging, mice were habituated to the treadmill under head-fixed conditions for 40 min per day over 2 weeks. Ca^2+^ imaging was performed using a two-photon microscope (Zeiss LSM 7 MP, Carl Zeiss, Jena, Germany) equipped with a water immersion objective lens (Apochromat 20, NA = 1.0, Carl Zeiss). Two-photon excitation at 900 nm for GCaMP6s imaging was carried out using a mode-locked Ti:sapphire laser system (Chameleon, Coherent, USA). Data were acquired using ZEN software (Zeiss Efficient Navigation, Carl Zeiss) at 4.4 Hz for imaging of S1 and 32 Hz for imaging of the cerebellar Bergmann glia.

### Motion tracking during Ca^2+^ imaging

Mouse locomotion was recorded using a video camera. Motion tracking was performed by a custom program written in LabVIEW (National Instruments, USA) and was synchronized with two-photon imaging by a trigger generated in the program. Mouse locomotion was recorded at a rate of 64 Hz using a high-speed CCD camera (IPX-VGA210, IMPERX, USA) with infrared illumination (DR4-56R-IR85, LVS, S. Korea). The recorded video had 64 frames per second, with a frame size of 720 × 480 pixels. To assess the level of locomotion, the difference in intensity of each pixel between frames was calculated and summarized across all the pixels. If the summarized value of a frame exceeded an arbitrary threshold determined by an experimenter blinded to the treatment information, the frame was scored as a locomotion-positive frame.

### Experimental models of spontaneous pain

For the formalin-induced pain model, 10 μl of formalin (5% or 1%) or vehicle solution was injected into the right hind paw. For the capsaicin model, 10 μl of capsaicin (0.01%) was delivered to the right hind paw. For the Complete freund’s adjuvant (CFA) model, 10 μl of CFA was injected subcutaneously into the plantar surface of the right hind paw. For the chemotherapy-induced peripheral neuropathy model, oxaliplatin (6 mg/kg) or 5% glucose was administered intraperitoneally. For the neuropathic pain model, PSL surgery was performed on the right hind paw under isoflurane anesthesia. The right sciatic nerve was exposed at the upper thigh of the mouse, and the nerve was ligated to a diameter of one-third to one-half of the original value with a 9–0 suture. In the assessment of analgesic effects, GB (100 mg/kg) and VX (50 mg/kg) were coadministered to the PSL animals 3 and 10 d after surgery. Imaging and behavioral tests were performed 30 min after the intraperitoneal injection of the drugs. For the itch model, chloroquine (100 μg/10 μl) was delivered to the right hind paw. Imaging was performed 1–5 min after chloroquine administration.

### Extracting Ca^2+^ traces and event detection

The imaging data were motion corrected using the Turboreg algorithm (Biomedical Imaging Group, Swiss Federal Institute of Technology, Lausanne, Switzerland). Regions of interest (ROIs) were detected using the CNMF-E algorithm^25^ and then manually reviewed. Spatial information on the ROIs was imported into ImageJ (https://imagej.nih.gov/ij/), and the average fluorescence in each ROI was calculated along with the frame. The events were detected from the extracted Ca^2+^ traces using MLSpike^26^, an open-source algorithm. The hyperparameters for MLSpike were set as follows: *a* = 0.3, tau = 1, saturation = 0.1, finetune.sigma = 0.02, and drift.parameter = 0.1. The drift signal from MLSpike was used to calculate the amplitude and frequency of the events.

### Ca^2+^ activity normalization

A Gaussian window^27^ was applied to reduce the noise of the extracted Ca^2+^ traces. The average value of the activity below the 70th percentile in each ROI was used as the baseline fluorescence activity (F_0_). All activity signals were transformed to dF/F_0_ in each ROI to normalize the scale range. The length of the data sequence was fixed at 497 frames. When the length of the sequence exceeded 497 frames, the data were divided using the window slicing method (window size of 497 frames, step size of 10 frames).

### Preprocessing for deep learning and deep learning architecture

The Ca^2+^ imaging data obtained in one imaging session had a size of n × m, where n was the number of ROIs and m was the number of frames (typically 497 frames for 2 min). The deep learning model was trained to have an output of [1, 0] for the non-pain condition or [0, 1] for the pain condition. The neural network was implemented using Keras^28^. The input data had dimensions of (k, 497, 1), where k is the number of input data sequences in the training set, 497 is the length of each data sequence, and 1 is the number of features. This input was forwarded to bidirectional long short-term memory recurrent neural networks^29^ activated by a hyperbolic tangent function. Then, the data were fed to two dense layers that had dropout rates of 0.2 and 0.1. These two dense layers were activated according to rectified linear unit (ReLU)^30^ and sigmoid activation functions, respectively. Finally, the data were fed to a dense layer that was activated by the softmax function, which is defined as follows:

1 $$S\left( {y_i} \right) = \frac{{e^{y_i}}}{{\mathop {\sum}\limits_i^n {e^{y_i}} }}$$

where y is the activated value, i is each node (i.e., class), and n is the total number of nodes or classes. The loss function was defined as follows:

2 $$- \frac{1}{N}\mathop {\sum}\limits_i^N {\left[ {y_i\log \hat y_i + \left( {1 - y_i} \right)\log \left( {1 - \hat y_i} \right)} \right]}$$

where y-hat is the estimated value that was activated by the softmax function, y is the label [1,0] for non-pain or [0, 1] for pain, and N is the total number of samples. The Adam optimizer^31^ was used, and the hyperparameters were set to the default values (lr = 0.01, decay = 1e-8, β1 = 0.9, β2 = 0.999). The L2 regularizer was applied to each dense layer to prevent overfitting. Thus, the loss function was redefined as follows:

3 $$- \frac{1}{N}\mathop {\sum}\limits_i^N {\left[ {y_i\log \hat y_i + \left( {1 - y_i} \right)\log \left( {1 - \hat y_i} \right)} \right]} + \lambda \mathop {\sum}\limits_i^N {W^{2_i}}$$

where W is the summation of the weight values of dense layers and λ is the arbitrary weight of the L2 regularizer. The initial weights of all layers were set based on the He uniform variance scaling initializer with a fixed random seed to ensure reproducibility.

### Pain classification deep learning model based on S1 activity

Ca^2+^ activity of the S1 in formalin-induced pain states was used as ground-truth data for pain. All baseline data were used as ground-truth data for non-pain. The quantity of pain data for deep learning training was less than the quantity of non-pain data. To balance the class sizes, the pain data were duplicated. To validate the deep learning model, we used the leave-one-subject-out (LOSO) cross-validation (CV) method. All data obtained from one mouse (subject) were isolated and assigned to the test session. Non-formalin data (i.e., capsaicin-, CFA-, PSL-, and oxaliplatin-induced pain data) were used in the test session directly because these data were never used in the training session.

### Pain classification deep learning model based on cerebellar glial cell activity

The Ca^2+^ activity of the cerebellar Bergmann glia in the capsaicin-induced pain state was used as ground truth data for pain. All baseline data were used as ground-truth data for non-pain. The Ca^2+^ imaging data of the cerebellum Bergmann glia (19200 frames per subject) were downsampled to 1/40 (479 frames). The locomotor information was used as a second feature, such that the input to the neural network had the shape of (k, 479, 2). For the test, the LOSO-CV method was applied.

### Itch classification deep learning model based on S1 activity

The Ca^2+^ activity of S1 in the chloroquine-induced itch state was used as ground truth data for itch. All baseline data were used as ground truth data for non-itch. The validation of the itch classification model was performed with the CV method. To compare itch and pain signals in S1, we used itch data as a test set for the pain classification deep learning model trained with formalin-induced pain signals.

### Statistics

**Fig. 1:**
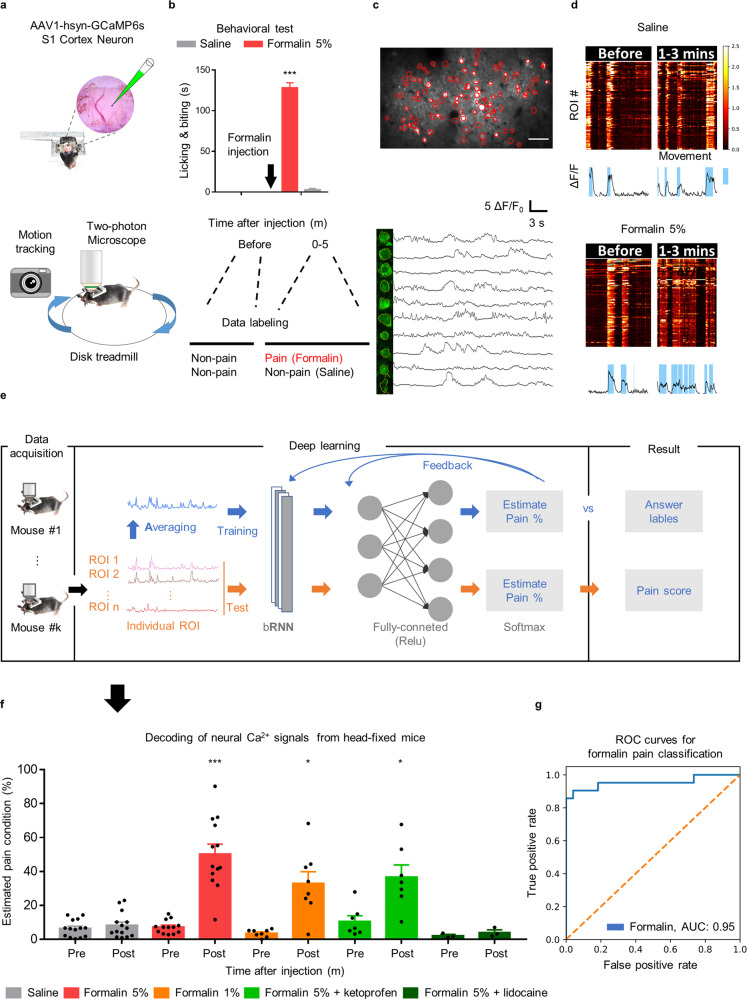
Conceptual framework and validation of AI-bRNN. **a** Schematic diagram of the experimental approach. AAV1-hsyn-GCaMP6s was injected into S1. **b** Imaging was performed for 2 min at each time point (before and 1–3 min after formalin injection). The time points for imaging were selected based on the levels of nociceptive behavior after formalin injection in freely moving animals. **c** A representative image of S1 neurons identified by semiautomated ROI analysis (top). Example Ca^2+^ traces from each ROI (bottom). The scale bar represents 50 μm. **d** Heatmaps showing the activity of S1 neurons. The line traces below each heatmap indicate the averaged values of all ROIs. The periods of mouse locomotion identified by the motion tracking analysis are overlaid on the line traces using sky-blue shading. **e** Architecture of AI-bRNN. The Ca^2+^ traces extracted from each ROI were averaged subject by subject to train the neural network. In the test session, the Ca^2+^ traces from individual ROIs were separately applied to the deep learning model for testing. **f** The predictions of AI-bRNN regarding whether the subject was experiencing pain. On the *x*-axis, ‘B’ indicates the time before injection. Saline (s.c.) group (*n* = 14 mice); formalin 5% (s.c.) group (*n* = 13 mice); formalin 1% (s.c.) group (*n* = 8 mice); formalin 5% (s.c.) + ketoprofen (100 mg/kg, i.p.) group (*n* = 7 mice); formalin 5% (s.c.) + 2% lidocaine (10 μl, s.c.) group (*n* = 3 mice). **g** The classification performance for formalin pain conditions based on the S1 neuronal signals. Scatter plots indicate individual data. Bars indicate the mean ± SEM; N.S., nonsignificant; ***P < 0.001, *P < 0.05 compared to the pre-injection period (Wilcoxon test).

Statistical analyses were performed using GraphPad Prism 7 (GraphPad Software, Inc.) or Python (SciPy library^32^). Two-factor repeated-measures ANOVA and Student–Newman–Keuls post hoc tests were used in Supplementary Fig. 6a, b. Pearson correlation analysis was used in Supplementary Fig. 3b and Supplementary Fig. 4c. The Wilcoxon test was used in Fig. 1f. The Mann–Whitney *U* test was used in other analyses. All values are represented as the means ± SEM.

## Results

### AI-bRNN detects information on formalin-induced spontaneous pain and the efficacy of painkillers from S1 neuronal Ca^2+^

To identify a prototype of neuronal activity patterns in spontaneous pain, we recorded S1 neuronal Ca^2+^ activity during formalin-induced spontaneous pain in awake, head-fixed mice (see Methods for details). Using in vivo two-photon microscopy and the genetically encoded Ca^2+^ indicator GCaMP6s, we imaged the Ca^2+^ activity of layer II/III neurons in the left S1 of head-fixed mice while tracking their motion using an infrared camera (Fig. 1a). Consistent with previous reports^33^, pain behaviors (i.e., licking and biting) were most prominent at 0–5 min after a formalin injection (5%, 10 μl, s.c.) into the right hind paw in freely moving mice. Only the Ca^2+^ activity signals recorded 1–3 min after the formalin injection were used as ‘pain’ condition signals (Fig. 1b). We extracted raw Ca^2+^ traces from each region of interest (ROI) (Fig. 1c) and matched the heatmap-visualized Ca^2+^ traces (Fig. 1d, top) and the averaged trace (Fig. 1d bottom) with the motion tracking data (blue background, Fig. 1d, bottom).

However, using conventional Ca^2+^ analyses^26^, we could not collect pain-specific information from S1 neurons. The amplitude and frequency of Ca^2+^ events showed no differences between the pain and non-pain conditions (Supplementary Fig. 1a, b), and the receiver operating characteristic (ROC) curve showed poor AUC scores of 0.61 and 0.55, respectively (Supplementary Fig. 1c). These negative results may not be surprising, considering that S1 neurons process touch and proprioception as well as pain^16,19^. Hence, we developed a deep learning algorithm to detect distinct features of Ca^2+^ activity that represent spontaneous pain.

Given the complexity of biology-driven data, adequate preprocessing steps are critical for the performance of deep learning model algorithms^34^. Indeed, when data were fed to the deep learning model without configuration of data preparation, the results showed poor performance (i.e., training and testing with individual ROI activity signals) (Supplementary Fig. 2a–c). Thus, we developed optimized preprocessing steps through trial and error and decided to use simplified data (sequential averaged activity signals across the ROIs) for the training session and individual data (sequential activity signals from each ROI) for the test session (Fig. 1e and Supplementary Fig. 2a–c). Since the formalin pain model exhibits clear, strong, and measurable pain behaviors^35^, we utilized S1 neuronal Ca^2+^ activity signals recorded at 1-3 min following formalin injection as supervisory signals for AI-bRNN. After training the AI-bRNN, we tested its classification performance with the LOSO-CV method. AI-bRNN predicted spontaneous pain conditions depending on the formalin concentration (5%, 1% and saline; Fig. 1f, g). Mild (ketoprofen, 100 mg/kg, i.p.) and strong (2% lidocaine, s.c.) painkillers reduced the estimated pain values in the 5% formalin group to the levels of the 1% formalin group and a saline-treated non-pain group (Fig. 1f). All of these pain values estimated by AI-bRNN in head-fixed mice were similar to the pain behaviors measured in freely moving mice (Supplementary Fig. 3a), with a highly positive correlation between the two (*r* = 0.97; Supplementary Fig. 3b). Along with the early phase of pain after formalin injection, we examined the late phase of pain. The results showed a late phase of pain that was distinct from the preceding period, and the estimated pain level was reduced to zero by injection of ketoprofen (Supplementary Fig. 4a, b). To compare the analgesic effects of ketoprofen between the early and late phases, we normalized the pain level to 1 for each phase. Ketoprofen showed a significantly stronger analgesic effect in the late phase than in the early phase (Supplementary Fig. 4c). The early phase of formalin pain is the result of a direct effect on nociceptors, and the late phase is an inflammatory response^36^. Therefore, the observation that ketoprofen, an anti-inflammatory drug, had a stronger analgesic effect in the late phase than in the early phase is consistent with a previous report. Together, these results indicate that our deep learning model can quantify the intensity of spontaneous pain and evaluate the efficacy of analgesics.

### AI-bRNN can be applied to other clinically relevant pain models, including chronic neuropathic pain

**Fig. 2:**
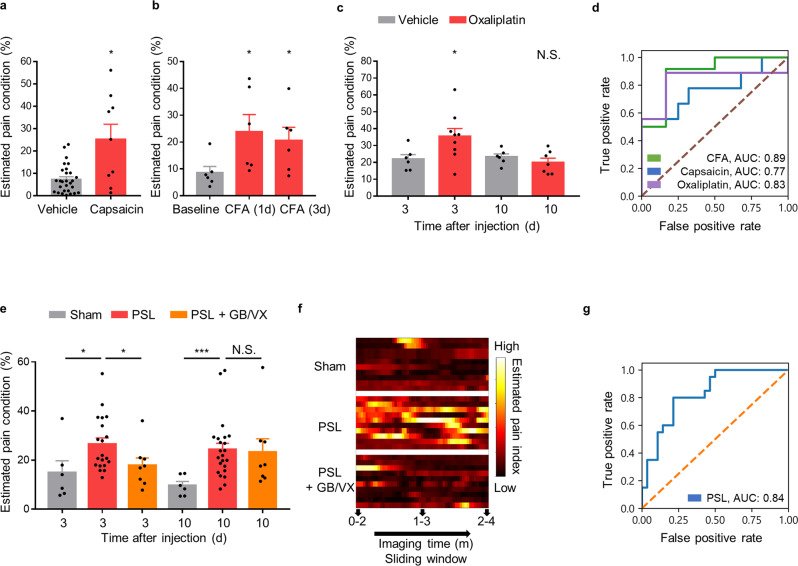
Broad applicability of AI-bRNN to various pain models with different chronicities. Estimated pain values of the **a** capsaicin-, **b** CFA-, and **c** oxaliplatin-injected animals. The estimated pain values are based on the Ca^2+^ activity of the neurons in S1. Saline (s.c.) group (*n* = 28 sessions from 14 mice); capsaicin (0.01%, 10 μl, s.c.) group (*n* = 9 mice); CFA (10 μl, s.c.) group (*n* = 6 mice); oxaliplatin (6 mg/kg, i.p.) group at 3 d (*n* = 9 mice); oxaliplatin group at 10 d (*n* = 7) **d** Classification performance in the capsaicin-, CFA- and oxaliplatin-induced pain conditions. **e** Estimated pain values of the animals subjected to PSL or sham surgery. Sham group (*n* = 6 mice); PSL group at 3 d (*n* = 20 mice); PSL group at 10 d (*n* = 24 mice); PSL + GB/VX (GB 100 mg/kg, VX 50 mg/kg, i.p.) group (*n* = 8 mice) **f** Heatmap plots showing changes in estimated pain values over time with 2-min time resolution. **g** The classification performance for PSL pain conditions based on the S1 neuronal signals. Scatter plots indicate individual data. Bars indicate the mean ± SEM; N.S., nonsignificant; ***P < 0.001, *P < 0.05 compared to baseline (Mann–Whitney *U* test).

Next, we tested whether AI-bRNN trained on S1 neuronal Ca^2+^ during formalin-induced pain is broadly applicable to various pain models with different chronicities. Our deep learning algorithm discriminated acute spontaneous pain induced by capsaicin (0.1%, s.c.) from the non-pain condition (Fig. 2a). AI-bRNN also distinguished subchronic spontaneous pain on Days 1 and 3 after injection of complete Freund’s adjuvant (CFA; 10 μl, s.c.) (Fig. 2b). We note that this could not be accomplished by the measurement of facial expression using GS^37^ or by analyzing locomotor behavior (Supplementary Fig. 5a, d) or the mean calcium activity of S1 (Supplementary Fig. 5b, e). Additionally, the pain index estimated by AI-bRNN was not correlated with S1 calcium activity or movement (Supplementary Fig. 5c), indicating that the deep learning model detects a feature distinct from body movement and the simple intensity of S1 calcium activity.

In addition to CFA-induced pain, AI-bRNN detected subchronic pain conditions in a mouse model of chemotherapy-induced peripheral neuropathy induced by oxaliplatin treatment (6 mg/kg, i.p.). The pain value estimated by AI-bRNN was significant at 3 d but returned to the control level at 10 d after oxaliplatin injection (Fig. 2c), consistent with the previously known symptomatic time course of this model^38^. These results indicate that AI-bRNN can discriminate capsaicin-, CFA- or oxaliplatin-induced pain from the non-pain condition, although the model was trained exclusively with data from mice with formalin-induced pain (Fig. 2d).

The diagnosis and treatment of spontaneous pain in neuropathic pain conditions have been a major challenge for pain researchers for decades. The previous approach using the GS could not detect pain in a chronic neuropathic pain model. Another method, CPP, merely detected the existence of an aversive state suggestive of spontaneous pain in model animals using pharmacological intervention that involves the reward system. Our approach using AI-bRNN could identify the condition of spontaneous pain in neuropathic pain mice at the early (3 d) and late (10 d) phases following PSL injury (Fig. 2e–g) without the use of pharmacological intervention. We also applied a sliding window method (segmentation of estimated pain values by 2-min time windows) and tracked the sequential change in the estimated pain values. Using this technique, we revealed when, how long, and at what degree of severity PSL group mice felt spontaneous pain (Fig. 2f, Supplementary Fig. 6a–c). We then asked whether our method could be used to measure the efficacy of analgesic drugs against spontaneous neuropathic pain. Combined treatment using gabapentin (GB; 100 mg/kg, i.p.) and venlafaxine (VX; 50 mg/kg, i.p.)^39,40^, which are clinically utilized analgesics, attenuated the estimated pain value at 3 d following PSL (Fig. 2e, f). Interestingly, however, the estimated pain value was not reduced when the same drugs were injected 10 d after PSL injury. These results resemble clinical studies that showed that the effects of these conventional analgesics diminish with the duration of neuropathic pain symptoms in patients^41,42^.

We confirmed using a conventional von Frey test that the treatment could relieve stimulus-evoked pain (mechanical allodynia) in both phases (Supplementary Fig. 7a, b). Taken together, our findings indicate that AI-bRNN has broad applicability to various animal models of spontaneous pain and emphasize that painkillers should be assessed separately for different pain types (i.e., evoked vs. spontaneous).

### Versatility of AI-bRNN

**Fig. 3:**
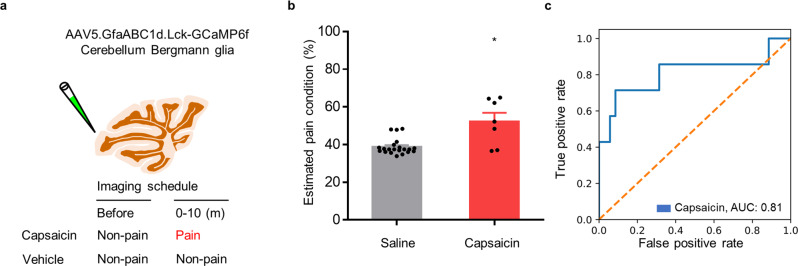
Classification performance of AI-bRNN in different brain regions and cell types. **a** Schematic drawing of the cerebellar Bergmann glia and the imaging timeline before and after capsaicin or vehicle injection. **b** Estimated pain values of capsaicin-injected animals. The estimated pain values were based on the Ca^2+^ activity of Bergmann glial cells in the cerebellum of the capsaicin group (*n* = 7 mice) and the saline group (*n* = 14 mice). The data from the baseline non-pain condition (before the capsaicin injection) were pooled with the data from the saline-injected animals. **c** The classification performance for the capsaicin-induced pain condition based on the cerebellum Bergmann glia signals. Scatter plots indicate individual data. Bars indicate the mean ± SEM; *P < 0.05 compared to the matched control group (Mann–Whitney *U* test).

We then tested the versatility of AI-bRNN by applying it to a different brain region (cerebellum) and cell type (glia), as well as to a different form of somatosensation (itch). Capsaicin-induced spontaneous pain information is transmitted to the cerebellum^43,44^, leading to increased neuronal activity and subsequent Ca^2+^ elevation in the resident Bergmann glia (unpublished data). We expressed GCaMP6f in the Bergmann glia of cerebellar cortex lobule IV/V to image Ca^2+^ activity during capsaicin-induced pain (0–10 min after injection) or non-pain conditions (Fig. 3a, see Methods for details). Since the boundaries of individual Bergmann glia are difficult to discern, we modified our deep learning model to use average Ca^2+^ activity from the whole imaging field, rather than individual Ca^2+^ traces from ROIs, in the test session as well as in the training session. Nevertheless, the algorithm robustly estimated the capsaicin pain condition (Fig. 3b, c).

**Fig. 4:**
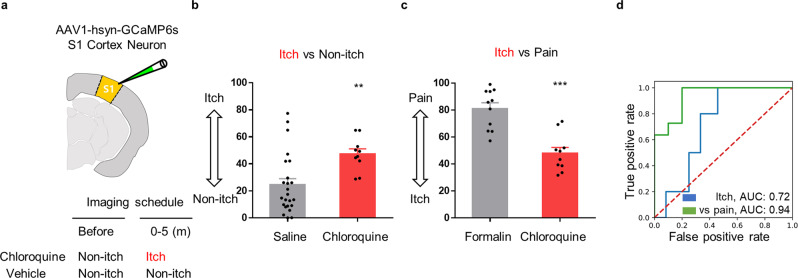
AI-bRNN can classify another somatosensation, itch. **a** A schematic drawing of the Ca^2+^ imaging schedule for S1 neurons in the chloroquine-induced itch conditions. **b**, **c** Estimated itch values of the chloroquine-injected animals based on the Ca^2+^ activity of S1 neurons. The data from the baseline non-itch condition (before chloroquine injection) were pooled with the data from the saline-injected animals. Saline (10 μl, s.c.) group (*n* = 24 mice); chloroquine (100 μg/10 μl, s.c.) group (*n* = 10 mice); formalin (5%, s.c.) group (*n* = 11 mice) **d** The classification performance for chloroquine-induced itch conditions based on the S1 neurons. Points on the scatter plots indicate individual data. Bars indicate the mean ± SEM; ***P < 0.001, **P < 0.01 compared to the matched control group (Mann–Whitney *U* test).

We also applied AI-bRNN to classify another somatosensation, itch, which shares common neuroanatomical pathways with pain while being a clearly distinct sensation^18^. We acquired S1 neuronal Ca^2+^ activity signals during chloroquine (100 μl, s.c.)-induced spontaneous itch (1–5 min after injection) or non-itch conditions (Fig. 4a) and used these signals as labeling data for the itch classification model. The results showed that the AI-bRNN approach could distinguish itch signals from non-itch signals (Fig. 4b, d). We also developed a deep learning model that differentiates itch from pain (Fig. 4c). Chloroquine-induced itch signals were used as labeled itch data (0 at the *y*-axis), and formalin-induced pain signals were reused as labeled pain data (100 at the *y*-axis); the established AI-bRNN clearly classified itch and pain (Fig. 4d). These results demonstrate the versatility of AI-bRNN.

## Discussion

Pain researchers and clinicians have long been eager to develop a real-time, scalable pain indicator. Since spontaneous ongoing pain is a major clinical problem and extremely difficult to treat, researchers have tried to develop intervention methods using various approaches in animal models. However, the successful translation of these methods into the clinic has yet to be achieved. In this regard, the lack of objective assessment of spontaneous pain in preclinical models has been regarded as a critical obstacle to filling the gap between animal experiments and clinical practice^8,10^. Here, we developed AI-bRNN, a deep learning algorithm for spontaneous pain assessment from brain activity signals obtained by two-photon microscopy in awake mice. We demonstrated that AI-bRNN robustly detects detailed information about spontaneous pain, such as its intensity, time point and duration, regardless of the phase (from acute to chronic) or the initiating cause of pain (inflammatory or neuropathic), and evaluates the analgesic efficacy of drugs. Thus, our unprecedented approach provides a powerful evaluation platform for spontaneous pain that will facilitate the successful translation of pain experiments into clinical practice.

The performance of our method is vastly superior to that of previous pain assessment tools that are based on observation of behaviors such as facial expressions^12^, place preference^13^, daily activities, food intake^45^ and ultrasound vocalizations^46^. First, AI-bRNN does not depend on behavioral responses; thus, there is no need to consider the motor function, learning ability, or the reward system of the animal. This would be an incomparable advantage in the study of pain symptoms in models of neurodegenerative diseases, such as Alzheimer’s disease^47^ or Parkinson’s disease^48^. Another advantage of AI-bRNN is that once the model is trained with pain datasets, testing new data requires minimal computing power and time. Therefore, real-time pain detection is entirely feasible (see Supplementary Movie 1) by applying the established model to the data acquisition step. This real-time analysis provides an advantage in studying the pharmacokinetics of drugs, enabling a better understanding of the intensity and duration of action of painkillers. Finally, AI-bRNN can be applied not only to neurons in the S1 but also to glia in other brain regions, such as the cerebellum (Fig. 3b, c). This multiregional applicability provides an additional benefit: when S1 is unavailable due to damage, signals from alternative brain regions could be used as pain indicators. These benefits that are obtainable through AI-bRNN cannot be achieved using existing methods, such as GS and CPP, which indicates that spontaneous pain detection based on neural activity has more sensitivity than behavioral responses.

Using AI-bRNN, the analgesic efficacy of drugs could be measured as well. The amelioration of pain by each drug treatment could be assessed in different types of pain models. Systemic administration of ketoprofen, a nonsteroidal anti-inflammatory drug, moderately reduced the pain value estimated by AI-bRNN, while subplantar injection of lidocaine strongly reduced the pain value in formalin-treated mice. All of these pain values determined by AI-bRNN in head-fixed mice were highly correlated with the pain behaviors measured in freely moving mice (^49,50^; see also Fig. 1f and Supplementary Fig. 3a, b). These results indicate that the values estimated by AI-bRNN can be used for quantification of pain and analgesic efficacy. This is also applicable to further experiments conducted in a mouse model of neuropathic pain. When GB/VX was administered 3 d after nerve injury, AI-bRNN detected clear analgesic effects of the treatment. On the other hand, AI-bRNN assessed that GB/VX exerted no significant analgesic effects when administered 10 d after nerve injury (Fig. 2e). This result contrasts with previous reports^39,40^ and our behavior experiments, which measured the analgesic efficacy of the drugs using a stimulus-evoked response test and observed a reduction in responsive behavior in animals (Supplementary Fig. 7a, b). Rather, the results from AI-bRNN resemble clinical studies reporting that the analgesic effects of the drugs on neuropathic pain are not reliable^51–53^, presumably because the drugs could not exert sufficient analgesic effects when administered to patients with chronic pain^41,42^. Based on these findings, we interpreted our method with AI-bRNN to represent the clinical situation more relevantly than the conventional stimulus-evoked method, and we propose that painkillers should be assessed separately for different pain types (i.e., evoked vs. spontaneous).

In the optimization process to establish pain detection with deep learning techniques, we considered various approaches in the configuration of data preparation. Data labeling is a critical part of the supervised learning process^34^, and inaccurately labeled data cause the deep learning model to perform poorly, as shown in Supplementary Fig. 2a–c. In this study, we injected formalin into the hind paw of mice to generate severe ongoing pain with a clear time window. The deep learning model showed high classification performance on various pain states when average ROI activity signals were used to train the model. In contrast, the performance was poor when the Ca^2+^ activity signals from individual ROIs were used as the pain label. These results might stem from the complexity in the S1 neuronal processing of sensory signals^54,55^. Considering that S1 neurons process multiple sensory signals, non-pain signals would be mixed into all the datasets we obtained. The individual non-pain signals included in the pain-label datasets would act as false pain data and interfere with the supervised learning. Averaging the Ca^2+^ activity of the ROIs would increase the signal-to-noise ratio, as information representing pain would be reflected by averaged activity in proportion to the pain signals included^56^. Using the averaged Ca^2+^ activity for training of the deep learning model would reduce the chance of training the model with irrelevant signals (false pain data). This explains the superior classification performance of the model trained with averaged Ca^2+^ activity compared to the model trained with individual Ca^2+^ activity.

In contrast to the training session, we found that the data configuration in the testing session was not crucial for the classification performance of the established model. Although the use of the AI setting (using the average signal for training and individual signals for testing) showed the highest performance among those that we tested (Supplementary Fig. 2a–c), sufficient classification performance was also obtained when the averaged signal from ROIs was used in the testing session (AA setting) (Supplementary Fig. 2a, b). Even using the average of the signals from the entire imaging field without ROI detection could achieve satisfactory classification performance (Fig. 3c). These results of testing with various settings provide additional flexibility to our deep learning approach. We believe that our approach provides a good example of a suitable optimization process for biology-driven data in supervised learning.

We adapted the bidirectional long short-term memory (LSTM) RNN, which is specialized for time-series data analysis, to the classification model^29^. bRNN has been reported to outperform other deep learning models that analyze time-series data^57,58^. The Ca^2+^ imaging data are also time series, and we expect that Ca^2+^ imaging data contain both forward and backward information. This deep learning architecture requires minimal field-specific knowledge because the feature extraction step is automated. For example, we adopted only the dF/F_0_ transformation, which is the procedure commonly applied to Ca^2+^ imaging. This minimized manual processing step promotes the versatility of AI-bRNN because it should be easily applicable to any type of Ca^2+^ data regardless of properties (e.g., event intensity, frequency and decay). We validated this versatility by pain estimation using glial Ca^2+^ activity recorded from the cerebellum (Fig. 3b, c). We also showed that AI-bRNN could be successfully applied to a distinct type of somatosensation, itch, which shares a neuroanatomical pathway with pain (Fig. 4b)^18^, and the model further distinguished between spontaneous pain and itch (Fig. 4c). Based on the flexibility, broad applicability and versatility of AI-bRNN, we strongly expect that our approach will be further extended to the detection of specific patterns not only from brain cellular Ca^2+^ activity but also from other forms of sequential data in the entire field of biology.

Recent technical advances have allowed us to record the activity of multiple neurons in the brain using electrocorticography^59,60^ or microelectrodes^61^ in humans. AI-bRNN can analyze the time-series data obtained with these techniques in real time. Such clinical applications of AI-bRNN, if combined with brain stimulation^62^, may enable simultaneous diagnosis and treatment of chronic pain in human patients, which is expected to reduce drug abuse, suicide rate and socioeconomic burden. In summary, AI-bRNN provides a new paradigm for the measurement of spontaneous ongoing pain and analgesic efficacy. This clinically relevant, scalable, real-time pain indicator paves the way for the successful translation of numerous mechanisms and analgesics discovered in preclinical animal models into pain relief in human patients.

## Supplementary information

Supplementary Figures 1–7

Supplementary Movie 1

## Data Availability

Python with the TensorFlow library was used to perform the analysis. All data and code associated with this study are presented in the paper, supplementary materials, or GitHub repository (https://github.com/KHUSKlab/paindecoding).
