# Supplementary material for: Development of a spontaneous pain indicator based on brain cellular calcium using deep learning
Source: Exp Mol Med. 2022 Aug 18;54(8):1179–87. doi: 10.1038/s12276-022-00828-7 (PMC9385425; doi:10.1038/s12276-022-00828-7)
Supplement: Supplementary file 1 — Supplementary Figures 1–7 [file 12276_2022_828_MOESM1_ESM.pdf]

Supplementary Materials:

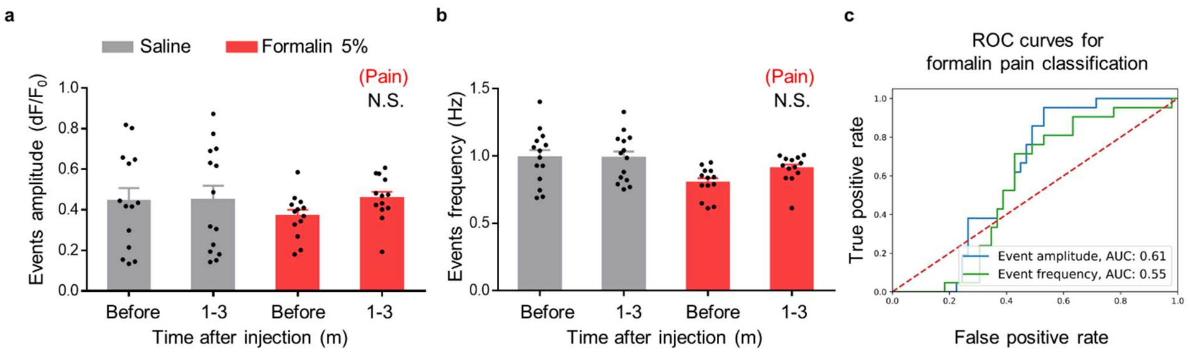

Supplementary Fig. 1. Limitations of conventional Ca^{2+} analysis in pain classification. (**a**) Mean amplitude and **(b)** mean frequency of Ca^{2+} events after formalin or saline injection. (**c**) Performance of the amplitude and frequency data for pain classification. Scatter plots indicate individual data. Bars indicate means ± SEM; N.S., non-significant; ***P < 0.001 compared to baseline.

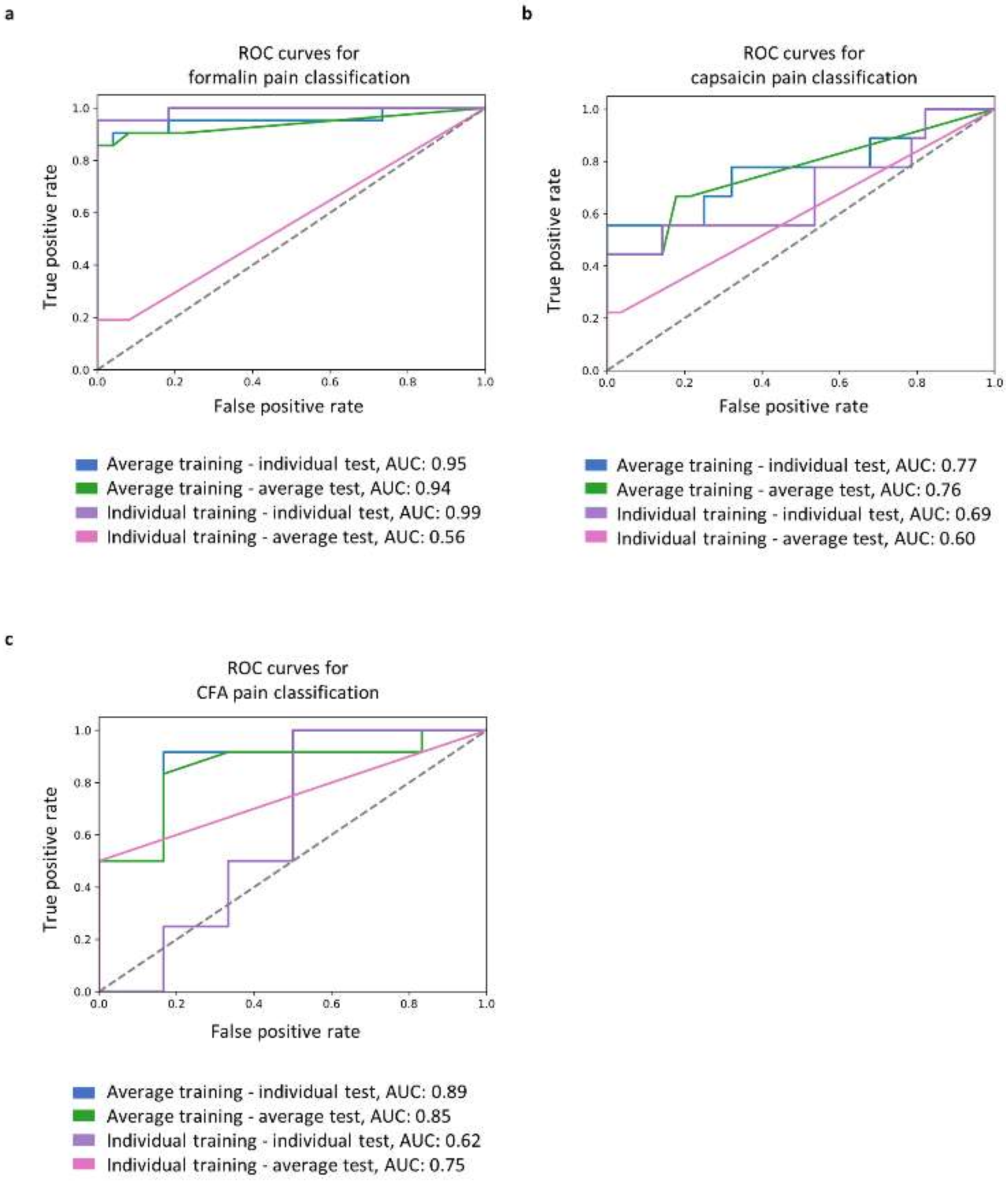

Supplementary Fig. 2. Classification performance of deep learning models with various preprocessings. Comparison of the area under the ROC curves for the four types of training-test strategies in each pain model. The classification performance for **(a)** formalin, **(b)** capsaicin or **(c)** CFA pain conditions based on the S1 neuronal signals.

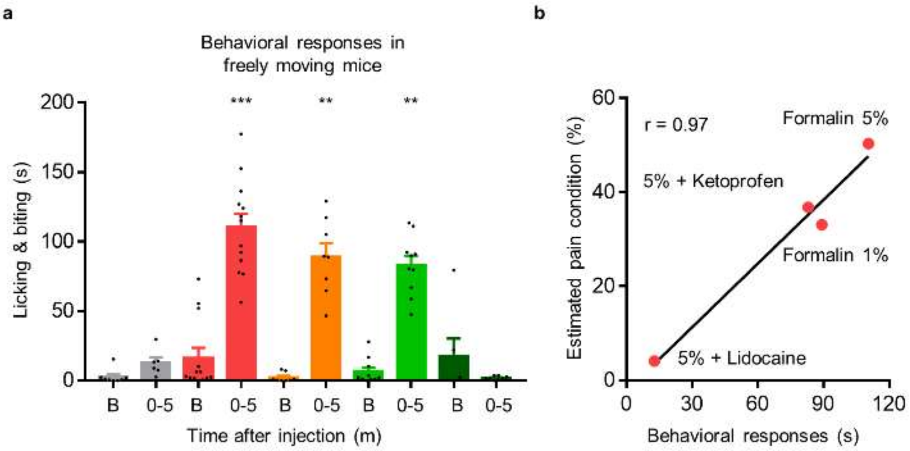

Supplementary Fig. 3. Correlation between pain prediction by AI-bRNN and behavioral responses in formalin-injected mice. (a) Nociceptive behaviors in freely moving mice. Saline group ($n = 8$ mice); 5% formalin group ($n = 13$ mice); 1% formalin group ($n = 8$ mice); 5% formalin + ketoprofen group ($n = 10$ mice); 5% formalin+ 2% lidocaine group ($n = 6$ mice) **(b)** Pearson's correlation between the group average of the estimated pain values and the behavioral response time (Pearson's r = 0.97). Scatter plots indicate individual data. Bars indicate means ± SEM; $^{***}P < 0.001$, $^{**}P < 0.01$ compared to baseline.

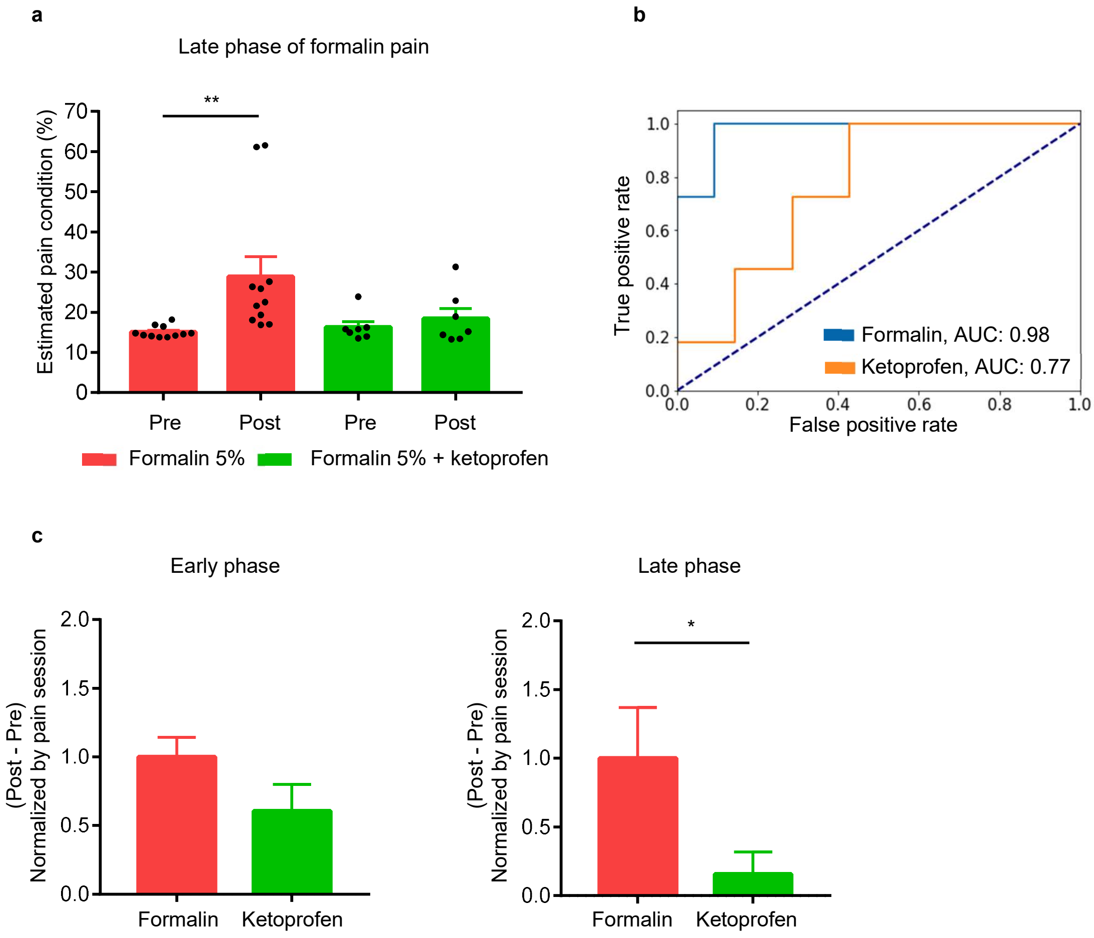

Supplementary Fig. 4. Application of AI-bRNN on the late phase of formalin-induced pain. **(a)** Estimated pain values of the formalin injected animal at late phases. Pre and post are defined by 8-10 minutes, and 18-20 minutes after formalin injection respectively. Formalin 5% (s.c.) group (n = 11 mice); formalin 5% (s.c.) + ketoprofen (100 mg/kg, i.p.) group (n = 7 mice). **(b)** Classification performances for formalin induced pain in the late phase from their pre periods and analgesic effects of ketoprofen in the late phase. **(c)** Comparison of analgesic effects of ketoprofen in early and late phases. To match the scale, the pain level in each phase was normalized to 1. Scatter plots indicate individual data. Bars indicate means ± SEM; *$P < 0.05$, **$P < 0.01$ compared to matched pre periods. (Wilcoxon test for paired data, Mann-Whitney test for unpaired data).

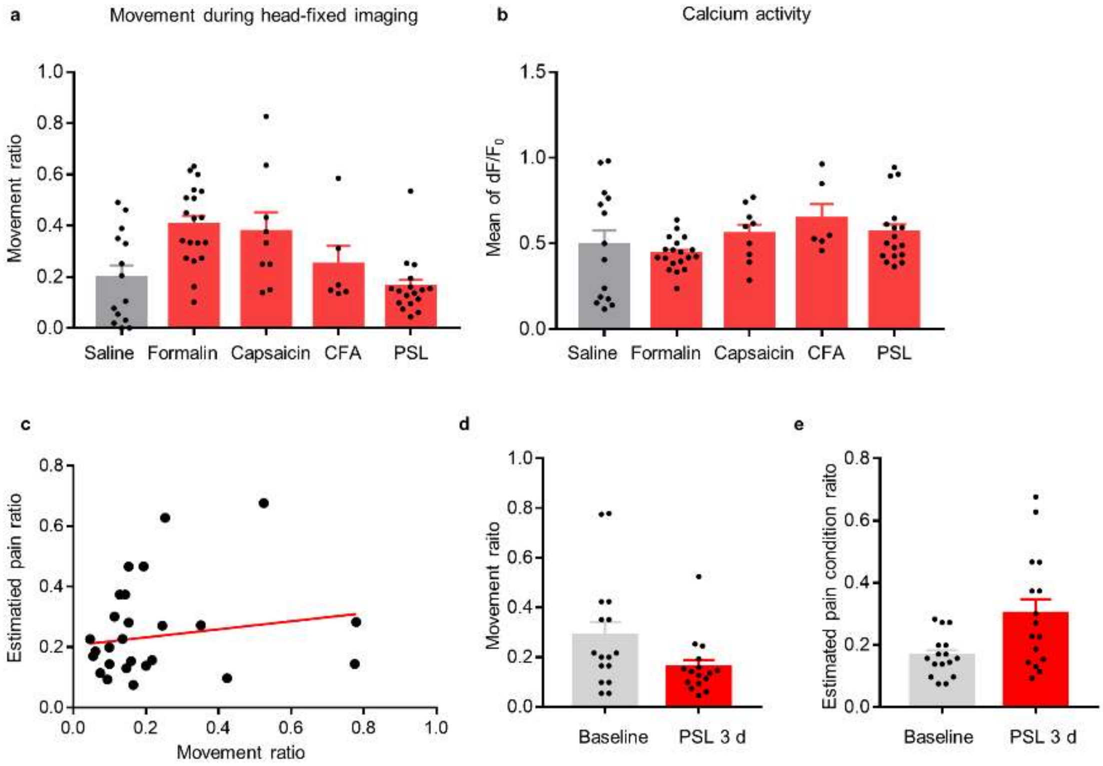

Supplementary Fig. 5. Estimated pain ratio can predict chronic pain state and are not correlated with movement or calcium activity. (a) Body movement of various pain models. **(b)** Mean of calcium activity of various pain models. Bars indicate means ± SEM. Scatter plots indicate individual data. *P < 0.05 compared to the matched non-pain group (Mann-Whitney test). **(c)** Black scatter plots show correlation between movement and estimated pain signals of mice in baseline or chronic pain state. Red line indicates linear regression of data. Pearson's correlation coefficient (r) is 0.1646. **(d)** Movement percentage and **(e)** estimated pain before and after nerve injury (*n* = 17 mice). Bars indicate means ± SEM.

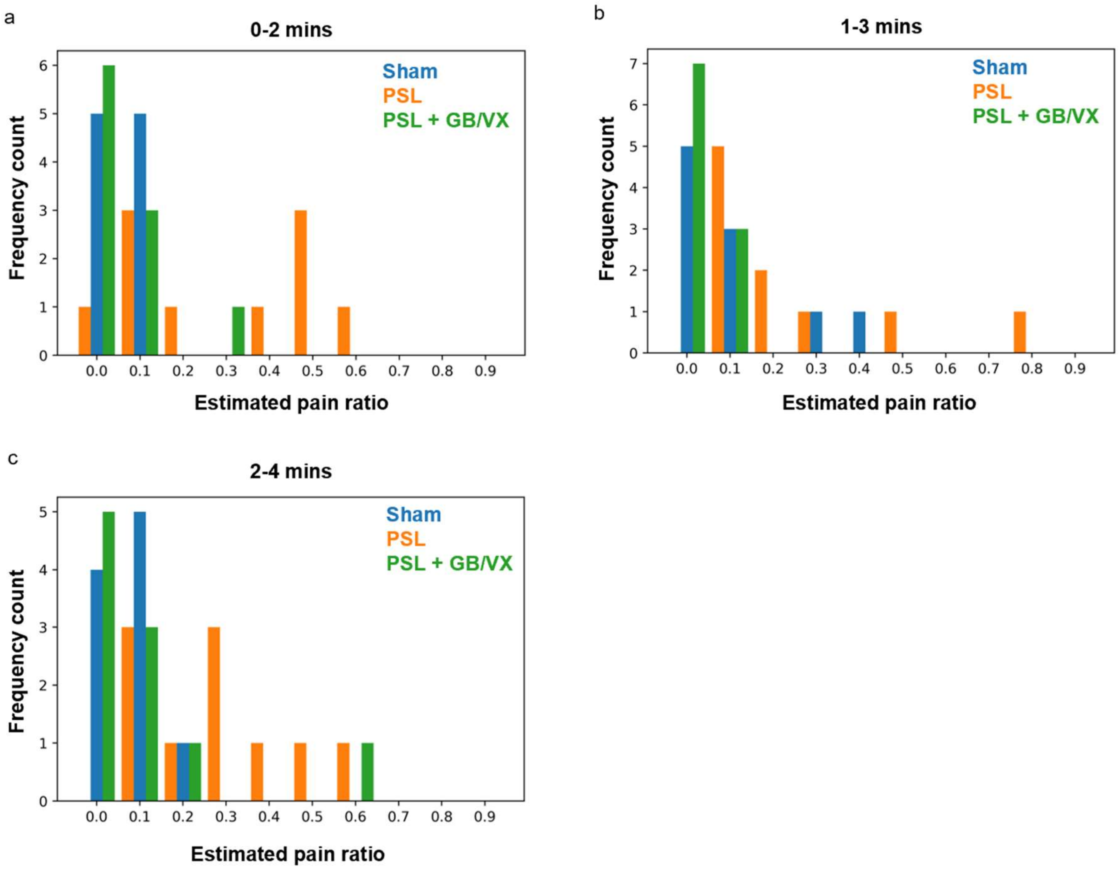

Supplementary Fig 6: Pain frequency histogram per sub-periods. Pain frequency histograms of estimated pain ratio. The x-axis indicates 0.1 intervals of data range from 0 to 1. Each histogram shows sub-period of **(a)** 0-2, **(b)** 1-3, or **(c)** 2-4 minutes.

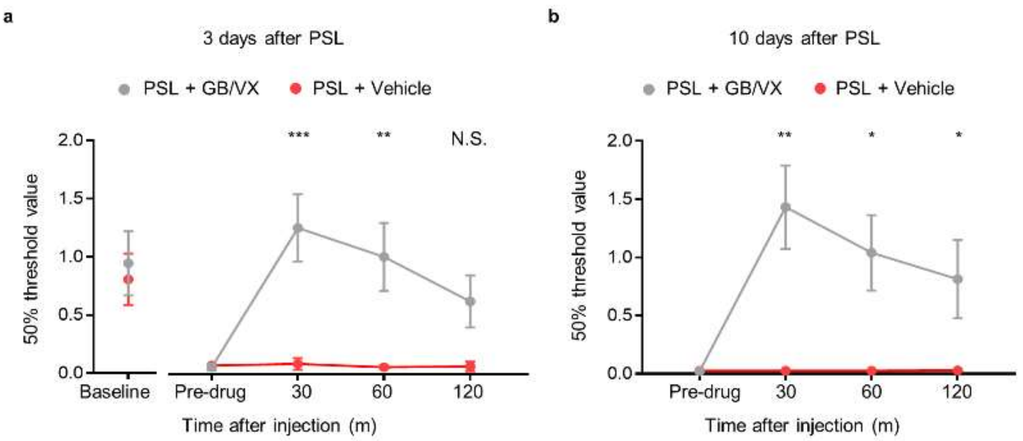

Supplementary Fig. 7. Relieving effects of GB/VX on neuropathic mechanical allodynia. Paw withdrawal threshold after GB and VX treatment on **(a)** days 3 and **(b)** 10 after PSL surgery in freely moving mice. Plots indicate means ± SEM; ***$P < 0.001$, **$P < 0.01$, *$P < 0.05$ compared to pre-drug values, N.S., non-significant; (two-factor repeated-measure ANOVA, Student-Neuman-Keuls post hoc test).
